# Limb Salvage Using Negative Pressure Wound Therapy and Split-Thickness Skin Grafting for Polymicrobial Ecthyma Gangrenosum in a Patient With Acute Lymphoblastic Leukemia: A Case Report

**DOI:** 10.7759/cureus.115919

**Published:** 2026-09-07

**Authors:** Andrea Nallely Garza-Cisneros, Rito Jafeth Medellin-Cristerna, Dayana Estefanía Gutierrez-Vega, Carolina Montenegro Jimenez, Sergio Miguel Suárez Fuentes, Martín Rodríguez-Garza, Samuel Hernandez-Pérez

**Affiliations:** 1 General Surgery, Hospital Regional de Monterrey, Instituto de Seguridad y Servicios Sociales de los Trabajadores del Estado (ISSSTE), Zapopan, MEX; 2 Plastic and Reconstructive Surgery, Hospital Regional Valentín Gómez Farías, Instituto de Seguridad y Servicios Sociales de los Trabajadores del Estado (ISSSTE), Zapopan, MEX; 3 Surgery, High Specialty Medical Unit 71, Torreon, MEX; 4 General Surgery, Hospital Regional Valentin Gomez Farías, Instituto de Seguridad y Servicios Sociales de los Trabajadores del Estado (ISSSTE), Zapopan, MEX; 5 General Surgery, Hospital Regional de Monterrey, Instituto de Seguridad y Servicios Sociales de los Trabajadores del Estado (ISSSTE), Monterrey, MEX

**Keywords:** acute lymphoblastic leukemia, limb salvage, negative pressure wound therapy, neutropenia, polymicrobial infection, split-thickness skin graft, tendon exposure

## Abstract

Ecthyma gangrenosum (EG) is a rare and potentially life-threatening cutaneous infection predominantly affecting profoundly immunocompromised patients. Although classically associated with *Pseudomonas (P.) aeruginosa*, polymicrobial infections may occur and can result in extensive tissue necrosis and limb-threatening defects. Reconstruction in severely immunocompromised patients is particularly challenging when exposed tendons are present. We present the case of a 32-year-old woman with relapsed B-cell acute lymphoblastic leukemia undergoing re-induction chemotherapy who developed profound neutropenia and rapidly progressive necrosis of the left dorsal hand and forearm following low-energy blunt trauma. The lesion evolved from a post-traumatic hematoma into a 20 × 8 cm full-thickness necrotic defect. Tissue cultures demonstrated polymicrobial infection with *P. aeruginosa, Acinetobacter baumannii complex, Stenotrophomonas maltophilia*, and *extended-spectrum β-lactamase-producing Escherichia coli*. Histopathology revealed full-thickness necrosis, dermal vascular thrombosis, and abundant intravascular Gram-negative bacilli, consistent with EG. Management included hematologic optimization, serial surgical debridement, targeted antimicrobial therapy, and negative pressure wound therapy at −125 mmHg. The extensor tendon paratenon was carefully preserved. After 14 days of negative pressure wound therapy and recovery of the absolute neutrophil count to 3.93 × 10³/µL, a healthy granulation bed developed, and definitive coverage was achieved with a split-thickness skin graft harvested from the ipsilateral thigh. Graft take exceeded 95%, with preservation of distal perfusion, sensation, and upper-extremity function. This case demonstrates that extensive polymicrobial EG can be successfully managed with a staged limb-salvage strategy integrating aggressive infection control, negative-pressure wound therapy, hematologic recovery, and delayed skin grafting. Notably, preserving the extensor tendon paratenon can enable successful split-thickness skin grafting in select patients with tendon exposure, thereby avoiding the morbidity of flap reconstruction.

## Introduction

Ecthyma gangrenosum (EG) is a rare but potentially life-threatening necrotizing cutaneous infection that predominantly affects immunocompromised patients. Although *Pseudomonas (P.) aeruginosa* is the pathogen most classically associated with the condition, EG may occur in the absence of bacteremia and may be caused by a broad spectrum of bacterial and fungal organisms [1-3].

The pathogenesis of EG is characterized by microbial invasion of the walls of dermal and subcutaneous blood vessels, resulting in vascular injury, thrombosis, luminal occlusion, tissue ischemia, and progressive full-thickness necrosis [2,4]. These mechanisms account for the rapid clinical progression and substantial morbidity associated with the disease.

EG occurs primarily in patients with impaired immunity, particularly those with hematologic malignancies and profound neutropenia. It has also been described in patients with other immunocompromising conditions, including diabetes mellitus and immunosuppressive therapy [1,2]. In patients with acute lymphoblastic leukemia undergoing intensive chemotherapy, prolonged neutropenia and mucosal barrier injury increase susceptibility to severe bacterial infections [5,6].

Historically, severe EG, particularly when associated with bacteremia or systemic infection, has been associated with substantial mortality. In addition to systemic complications, extensive tissue destruction may compromise limb viability and require amputation [2,7,8].

Negative pressure wound therapy (NPWT; V.A.C. Therapy, KCI, San Antonio, TX, USA) has become an important component of staged management for complex wounds after adequate infection control and surgical debridement. Its effects include continuous removal of wound exudate, reduction of edema, stabilization of the wound environment, and mechanical stimulation of granulation tissue formation [9,10]. These effects may facilitate progressive wound bed preparation for definitive reconstruction, including split-thickness skin grafting [11].

We present the case of a patient with relapsed acute lymphoblastic leukemia and profound neutropenia who developed extensive polymicrobial ecthyma gangrenosum of the upper extremity. A staged reconstructive approach consisting of serial debridement, NPWT, hematologic optimization, and delayed split-thickness skin grafting resulted in successful limb salvage and functional recovery. The case highlights the potential role of preserving the extensor tendon paratenon when planning definitive coverage of extensive tendon-exposed wounds.

## Case presentation

A 32-year-old woman with a history of B-cell acute lymphoblastic leukemia, initially diagnosed in November 2024, was admitted for re-induction chemotherapy after hematologic relapse was confirmed. Approximately two weeks before admission, she sustained a low-energy blunt trauma to her left upper extremity, resulting in the formation of a hematoma over the dorsal aspect of the hand and forearm.

Following initiation of chemotherapy, she developed severe pancytopenia and profound neutropenia. During this period, the hematoma progressively enlarged and evolved into a rapidly advancing necrotic lesion. The lesion initially presented with hemorrhagic bullae and violaceous erythema and subsequently progressed to a full-thickness necrotic eschar measuring approximately 20 × 8 cm, extending from the second through fifth metacarpophalangeal joints to the proximal third of the dorsoradial forearm (Figure 1).

**Figure 1:**
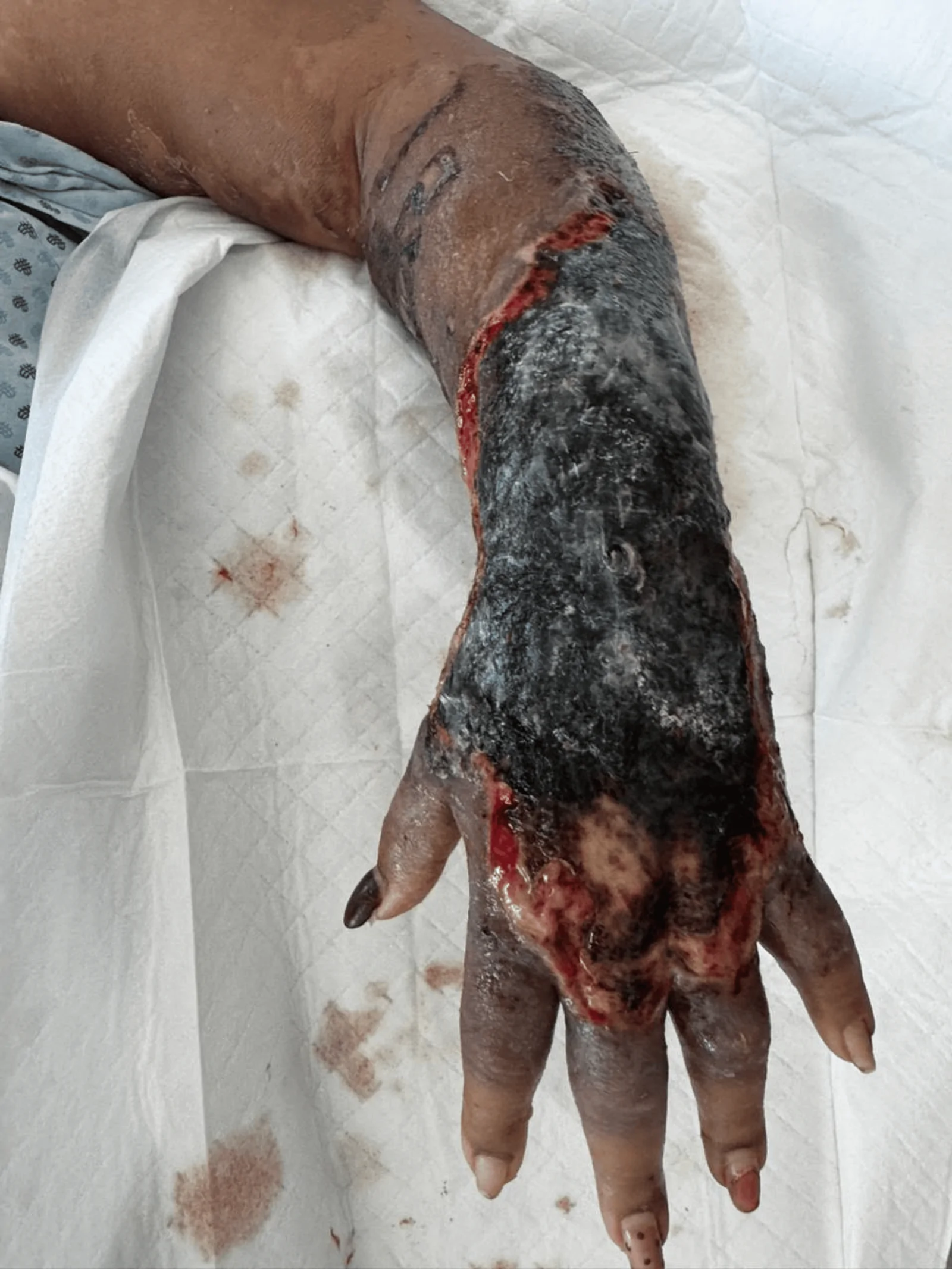
Initial presentation of polymicrobial ecthyma gangrenosum involving the left dorsal hand and forearm An extensive full-thickness necrotic plaque measuring approximately 20×8 cm extending from the second through fifth metacarpophalangeal joints to the proximal third of the dorsoradial forearm. Note the dark necrotic eschar, surrounding violaceous erythema, severe tissue edema, purulent exudate, and areas of epidermolysis and tissue separation at the wound margins.

At surgical evaluation, the patient was febrile but hemodynamically stable. Physical examination demonstrated severe edema of the left upper extremity, an extensive necrotic plaque with purulent exudate and foul-smelling areas of separation at the wound margins, and severe tenderness on palpation. Neurovascular examination revealed palpable radial and ulnar pulses, capillary refill of less than two seconds, and preserved sensation in the median, radial, and ulnar nerve distributions, with no clinical evidence of compartment syndrome.

Initial laboratory studies reflected profound chemotherapy-induced myelosuppression, with a hemoglobin level of 6.2 g/dL, total leukocyte count of 0.07 × 10³/µL, absolute neutrophil count of 0.05 × 10³/µL, and platelet count of 32 × 10³/µL. Following multidisciplinary management, hematologic recovery was achieved, with the absolute neutrophil count increasing to 3.93 × 10³/µL before definitive reconstruction (Table 1).

**Table 1:** Serial laboratory parameters across clinical stages of management Evolution of hematologic and inflammatory markers from initial admission during profound chemotherapy-induced neutropenia to post-NPWT wound bed optimization and final post-graft follow-up. Note the progressive recovery of the absolute neutrophil count and systemic stabilization prior to definitive split-thickness skin grafting. NPWT: negative pressure wound therapy

Laboratory Parameter	Critical Phase (Admission)	Post-NPWT Phase (Day 30 with NPWT)	Post-Graft Phase (Day 14)	Reference Range
Hemoglobin (g/dL)	6.2	8.8	10.2	12.0 – 15.5
Total Leukocytes (x10³/µL)	0.07	5.74	6.85	4.5 – 11.0
Absolute Neutrophil Count (ANC) (x10³/µL)	0.05	3.93	4.50	2.0 – 7.0
Platelets (x10³/µL)	32	268	245	150 – 450
Blood Urea Nitrogen / Creatinine (mg/dL)	64.2 / 1.20	10.2 / 0.46	11.0 / 0.60	7.0–20.0 / 0.6–1.1
Procalcitonin (ng/mL)	0.19	0.08	0.02	< 0.10

Intraoperative tissue cultures yielded polymicrobial growth of *P. aeruginosa*, *Acinetobacter (A.) baumannii* complex, *Stenotrophomonas (S.) maltophilia*, and extended-spectrum β-lactamase-producing *Escherichia (E.) coli*. Blood cultures were not obtained during the patient's hospitalization. Histopathological examination demonstrated full-thickness tissue necrosis, fibrin thrombi within dermal vessels, and abundant intravascular Gram-negative bacilli, findings consistent with ecthyma gangrenosum.

The diagnosis of ecthyma gangrenosum was supported by the combination of profound chemotherapy-induced neutropenia, a rapidly progressive necrotic cutaneous lesion, isolation of Gram-negative organisms from surgical wound cultures, and characteristic histopathological findings.

The lesion initially developed after blunt trauma and was initially considered a post-traumatic hematoma. However, during profound neutropenia, it rapidly progressed to hemorrhagic bullae, violaceous erythema, and extensive full-thickness necrosis. Histopathological examination demonstrated full-thickness tissue necrosis, fibrin thrombi within dermal vessels, and abundant intravascular Gram-negative bacilli. These findings, in the setting of severe immunosuppression and compatible microbiological results, supported the diagnosis of ecthyma gangrenosum rather than isolated traumatic tissue necrosis.

Surgical management

Treatment was performed using a staged reconstructive approach. Initial management focused on hematologic optimization through the transfusion of blood products to achieve adequate hemoglobin and platelet levels before surgery. This was followed by extensive surgical debridement, with complete excision of all necrotic tissue until healthy, bleeding tissue was encountered. Particular care was taken to preserve the paratenon of the extensor tendons, thereby maintaining a vascularized recipient bed suitable for subsequent reconstruction (Figure 2). Unfortunately, during surgical debridement, the extensor tendons were exposed and were partially affected by the necrotic process. Copious irrigation with normal saline was then performed, and tissue specimens were obtained for microbiological culture and histopathological analysis. At the time of definitive reconstruction, the wound was considered clinically suitable for split-thickness skin grafting.

**Figure 2:**
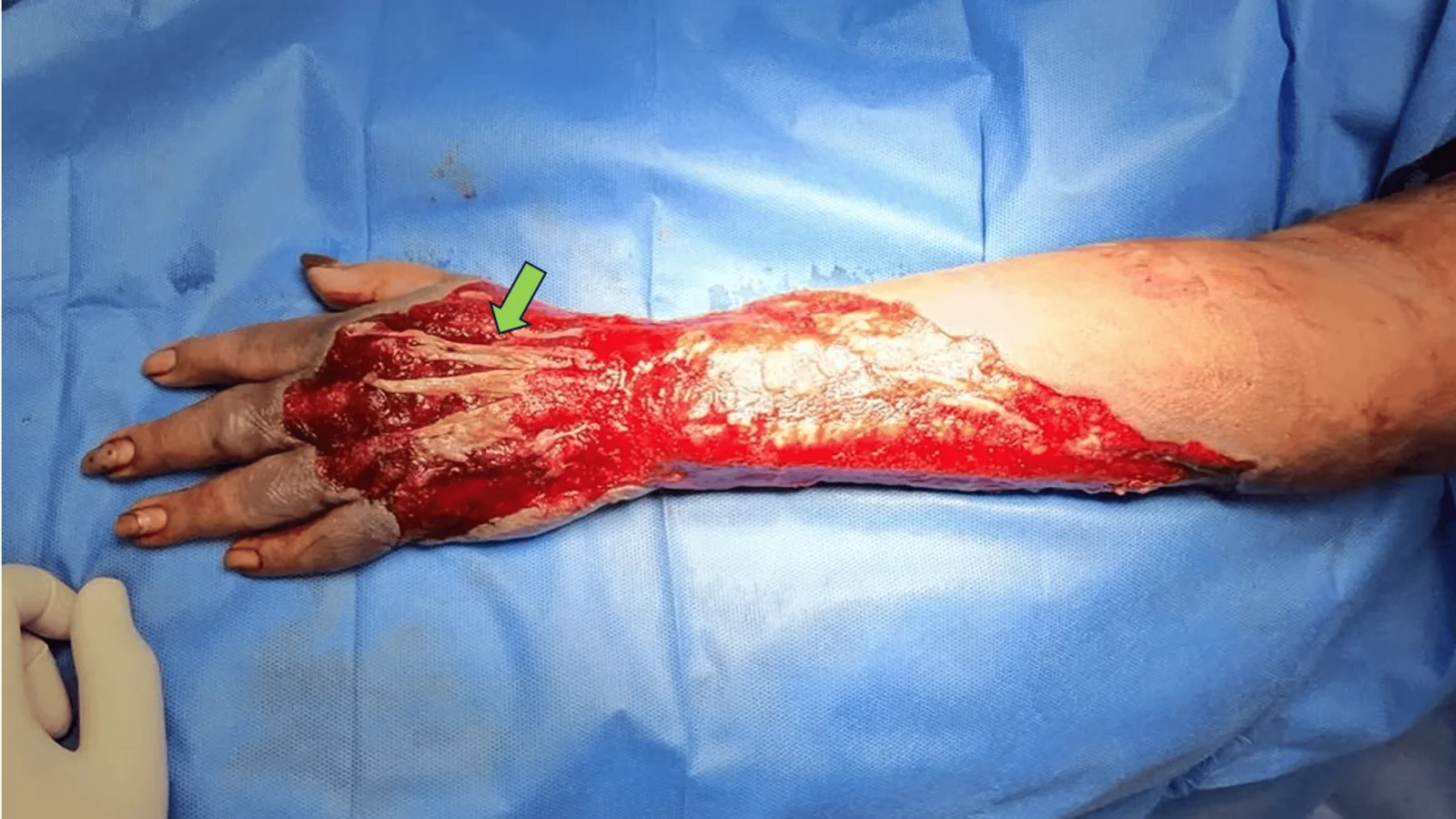
Wound bed appearance following radical surgical debridement Intraoperative view after complete excision of nonviable necrotic tissue, demonstrating a clean, vascularized wound bed over the left dorsal hand and forearm. Note the extensive defect with exposed extensor tendons over the dorsum of the hand (green arrow), with careful preservation of the intact, vascularized paratenon to serve as a recipient bed for subsequent reconstruction.

Once adequate local infection control had been achieved, NPWT was initiated using polyurethane foam with a nonadherent interface placed over the exposed extensor tendons on day 6. Subsequent V.A.C. changes were performed on days 10, 14, 18, 22, and 26. Continuous negative pressure of −125 mmHg was applied for 37 days, with 5 dressing changes performed in the operating room under sterile conditions (Figure 3). During this period, progressive reduction of edema and wound exudate was observed, accompanied by the development of a healthy, uniform granulation tissue bed without clinical evidence of persistent infection.

**Figure 3:**
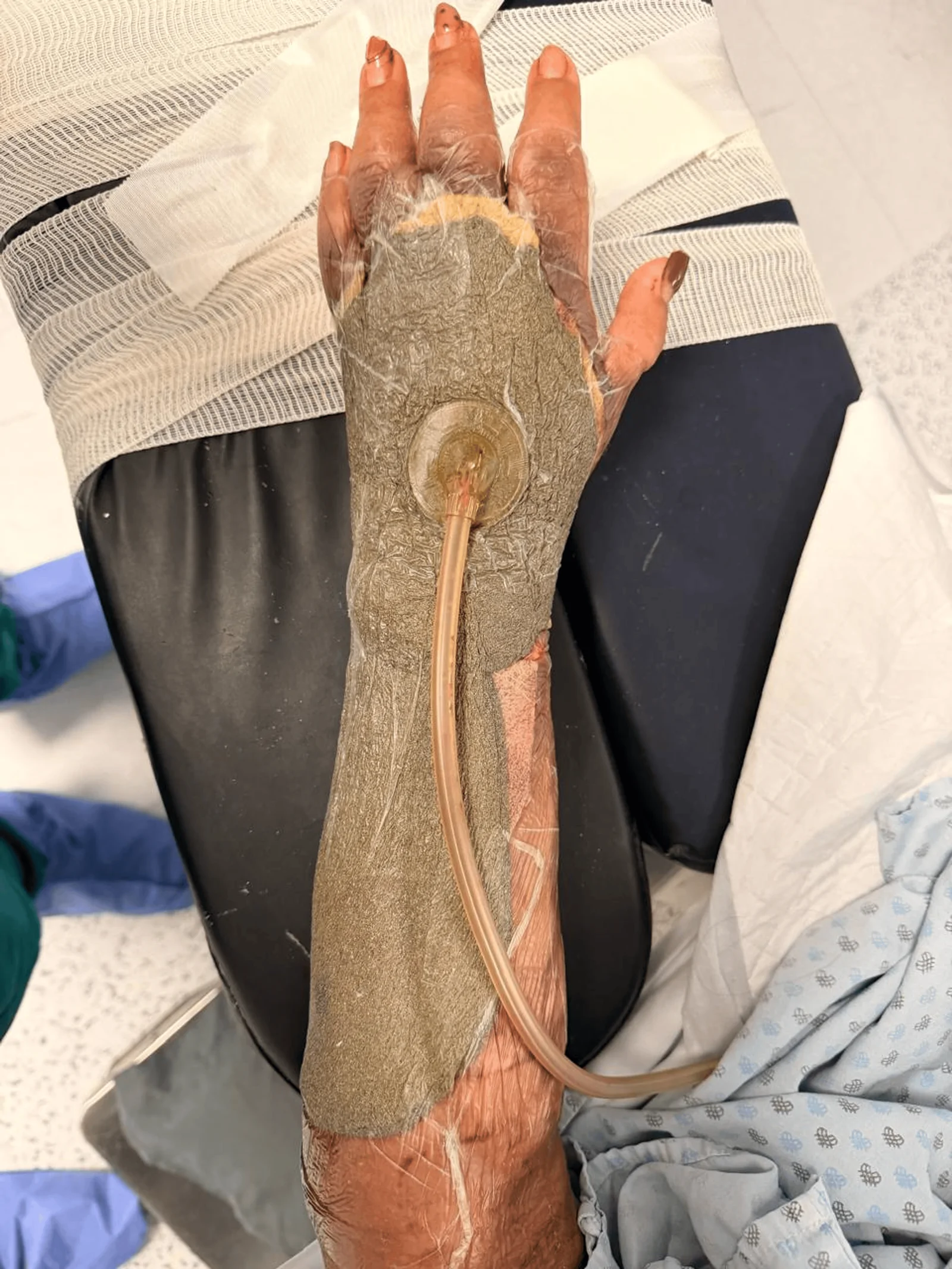
Negative pressure wound therapy (NPWT) on the left upper extremity The vacuum-assisted closure (V.A.C.) system was positioned over the left dorsal hand and forearm at a continuous pressure of −125 mmHg. A protective nonadherent interface was placed directly over the exposed extensor tendons beneath the polyurethane foam to preserve paratenon integrity, promote exudate drainage, and facilitate wound bed preparation prior to skin grafting.

The initial surgical wound culture grew *E. coli *and *P. aeruginosa*. *E. coli *was susceptible to Ertapenem, Meropenem, and Nitrofurantoin, whereas *P. aeruginosa* was susceptible to Ceftazidime, Cefepime, Ciprofloxacin, and Norfloxacin. A subsequent wound culture obtained 2 weeks later grew *S. maltophilia* and *A. baumannii *complex susceptible to Amikacin. Simultaneously, targeted antimicrobial therapy with Cefepime and Vancomycin was administered according to microbiological susceptibility testing for 14 days, together with granulocyte colony-stimulating factor, resulting in progressive recovery of the absolute neutrophil count.

Following complete infection control and adequate wound bed preparation, definitive reconstruction was performed using a split-thickness skin graft harvested from the ipsilateral anterolateral thigh at a thickness of 0.012-0.015 inches. The graft was meshed at a 1.5:1 ratio and secured with surgical staples and interrupted monofilament sutures (Figure 4). A volar forearm splint was applied to maintain the extremity in a functional position and protect the reconstruction.

**Figure 4:**
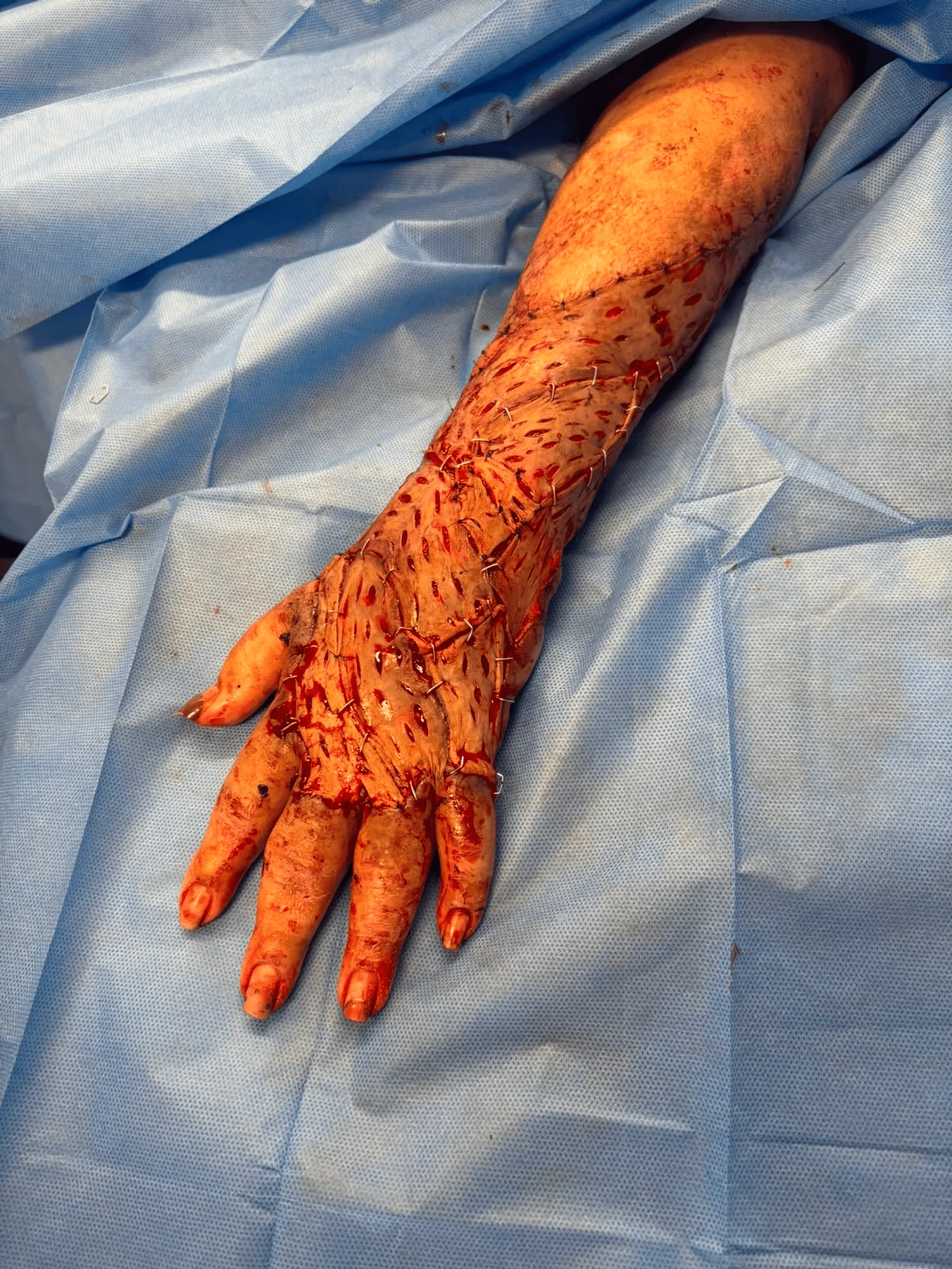
Immediate postoperative outcome following split-thickness skin grafting Definitive reconstruction of the defect on the left hand and forearm using a meshed split-thickness skin graft (1.5:1 ratio) harvested from the ipsilateral thigh. The graft was carefully adapted to the healthy granulation bed and secured along the wound margins and intermetacarpal spaces using surgical staples and interrupted monofilament sutures.

Split-thickness skin grafting was therefore selected as a less invasive reconstructive option in a patient recovering from profound chemotherapy-induced neutropenia and severe systemic immunosuppression. This approach avoided the additional operative complexity and donor-site morbidity associated with flap reconstruction.

Fourteen days after grafting, greater than 95% graft take was achieved based on clinical assessment at postoperative day 14, with excellent vascularization, complete healing of the donor site, and preservation of distal perfusion and sensation (Figure 5A). However, the patient had documented extensor tendon involvement and postoperative loss of hand extension. Formal measurements of range of motion, tendon excursion, or grip strength were not available. At outpatient follow-up one month after skin grafting, the graft demonstrated progressive maturation with complete preservation of the limb and no evidence of recurrent infection. Small residual areas of epidermolysis healed successfully with local wound care alone, without the need for additional reconstructive procedures (Figure 5B).

**Figure 5:**
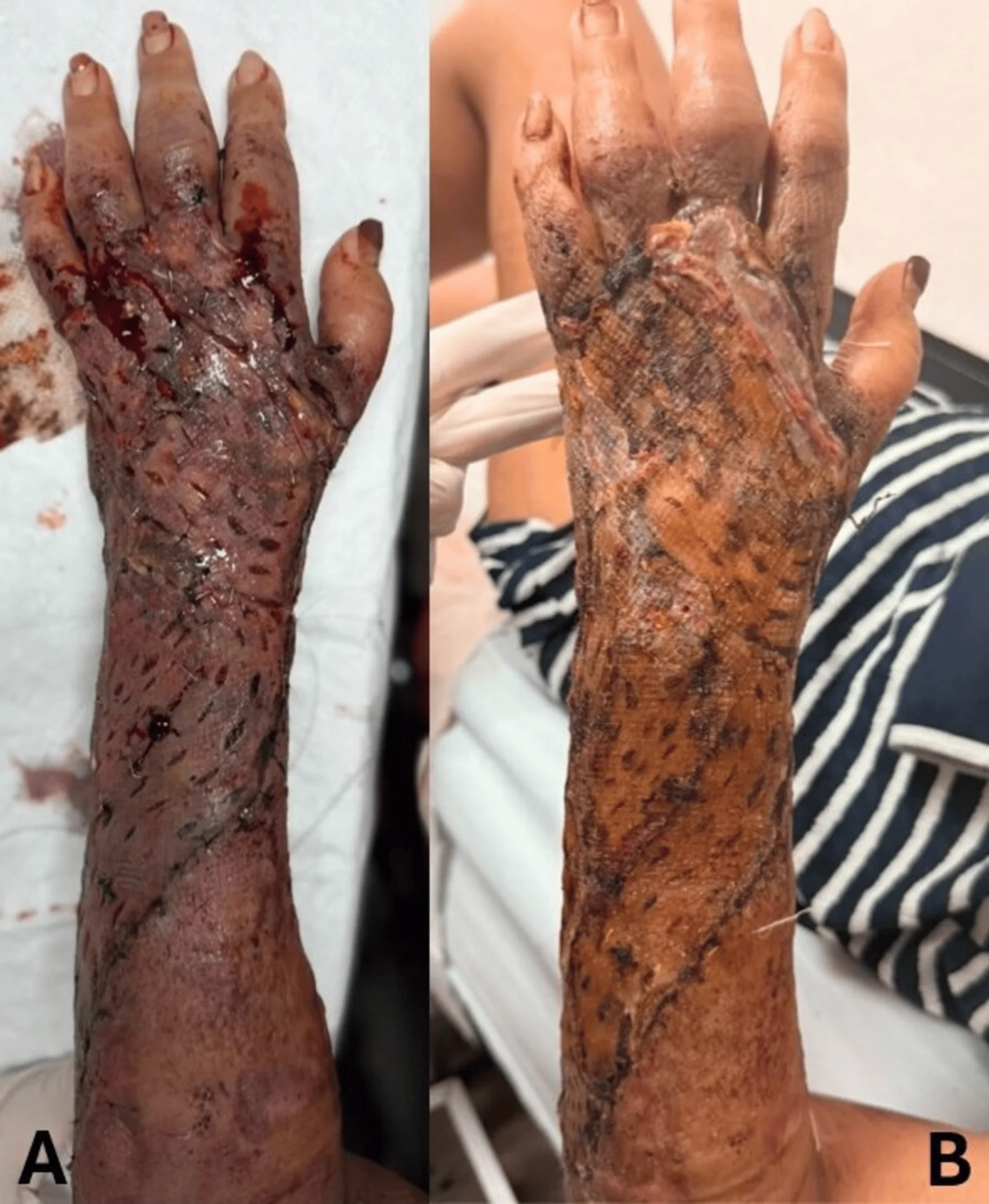
Postoperative progression and graft integration (A) Assessment 14 days after grafting showing greater than 95% graft take with adequate revascularization and viable integration over the recipient wound bed. (B) Outpatient follow-up one month after skin grafting, demonstrating advanced graft maturation and re-epithelialization. Note the complete preservation of distal perfusion, preservation of upper-extremity movement, and healing of minor superficial epidermolytic areas with conservative local wound care alone.

## Discussion

EG represents a particularly challenging clinical entity in profoundly immunocompromised patients because of its rapid progression, extensive tissue destruction, and the host's limited ability to contain infection [1,2]. Although *P. aeruginosa* remains the pathogen classically associated with EG, the disease has a broader microbiological spectrum that includes other Gram-negative bacteria, Gram-positive organisms, and fungi. Furthermore, EG may develop in the absence of bacteremia; therefore, negative blood cultures should not exclude the diagnosis when clinical and histopathological findings are suggestive [1-3].

The association between EG, neutropenia, and hematologic malignancies is well-established [1,2]. *S. maltophilia* has been described as an uncommon cause of EG in patients with leukemia and profound immunosuppression, while polymicrobial infections involving *P. aeruginosa* and *A. baumannii* have also been reported [12-14]. In the present case, the simultaneous isolation of *P. aeruginosa*, *A. baumannii* complex, *S. maltophilia*, and extended-spectrum β-lactamase-producing *E. coli* illustrates the broad microbiological spectrum that may be encountered in severely immunocompromised patients with EG.

The pathophysiology of EG largely explains the fulminant nature of the disease. Direct microbial invasion of dermal and subcutaneous vessels leads to vascular injury, thrombosis, tissue ischemia, and ultimately progressive full-thickness necrosis. Histopathological findings of vascular and perivascular microbial invasion are characteristic of the disease [2,4].

In profoundly neutropenic patients, impaired host defense may further facilitate disease progression [1,2]. In our patient, what initially appeared to be a benign post-traumatic hematoma rapidly evolved into extensive full-thickness necrosis involving the dorsum of the hand and forearm. This clinical course underscores the importance of maintaining a high index of suspicion for EG whenever progressive skin necrosis develops in neutropenic patients, even when an alternative explanation such as trauma initially appears plausible.

Surgical debridement

The primary objective of reconstruction in this setting is not immediate wound coverage but rather complete eradication of infection and devitalized tissue. Early and aggressive surgical debridement remains the cornerstone of local infection control in necrotizing soft tissue infections and should be repeated whenever residual nonviable tissue is identified [15,16]. A systematic review and meta-analysis demonstrated that early surgical debridement is associated with improved survival compared with delayed intervention [15].

However, debridement of a critically compromised upper extremity requires a careful balance between radical excision of necrotic tissue and preservation of reconstructive options. In our patient, meticulous preservation of the extensor tendon paratenon allowed maintenance of a vascularized wound bed despite extensive soft tissue loss. This decision was fundamental because it preserved the possibility of subsequent skin grafting and ultimately avoided the need for flap reconstruction.

NPWT as a bridge to definitive reconstruction

Following adequate infection control, NPWT served as an effective bridge between debridement and definitive reconstruction. Its role is not to replace surgical debridement but rather to optimize the wound environment through continuous exudate evacuation, edema reduction, wound stabilization, and mechanical stimulation of granulation tissue formation through macrodeformation and microdeformation [9,10,17,18].

In the present case, NPWT was maintained for 14 days, during which the wound area decreased by approximately 25%, while a healthy, well-vascularized granulation bed progressively developed. Importantly, this interval should not be viewed merely as a period of local wound management. It also provided time for systemic recovery, particularly restoration of neutrophil counts, before definitive reconstruction.

The literature also supports the use of NPWT following skin grafting. A systematic review and meta-analysis of randomized controlled trials demonstrated improved graft take and reduced reintervention rates compared with conventional dressings [11]. More recently, a meta-analysis of randomized controlled trials reported an approximately 8.3% absolute increase in overall graft take, together with lower rates of graft loss, postoperative complications, and reoperation when NPWT was used as an adjunct after skin grafting [19].

Taken together, these findings support the reconstructive sequence employed in our patient: radical debridement, temporary NPWT for wound bed optimization, and delayed definitive reconstruction once both local and systemic conditions had become favorable.

Split-thickness skin graft versus flap reconstruction

One of the principal reconstructive dilemmas in this case was whether the size of the defect and extensor tendon exposure mandated flap reconstruction.

Soft tissue defects of the hand and forearm with exposed tendons are generally considered challenging reconstructive defects, particularly when the tendon lacks an intact vascularized surface. Because a denuded tendon has limited intrinsic vascularity, it generally provides an inadequate recipient bed for skin graft survival. In contrast, preservation of the paratenon or development of a vascularized granulation layer over the tendon may provide a suitable recipient bed for a split-thickness skin graft [20].

In our patient, preservation of the paratenon during the initial debridement, followed by progressive wound bed optimization with NPWT, resulted in greater than 95% graft take. This outcome suggests that selected tendon-exposed wounds may be amenable to split-thickness skin grafting when an adequately vascularized recipient bed can be preserved or developed. Therefore, tendon exposure alone should not automatically mandate flap reconstruction; intraoperative assessment of paratenon integrity and wound bed viability should guide reconstructive decision-making [20].

This consideration is particularly relevant in patients recovering from intensive chemotherapy. Microsurgical reconstruction may involve longer operative times, greater physiological stress, and additional donor-site morbidity in patients with limited physiological reserve. Although vascularized flaps remain indispensable when critical structures are exposed or when the wound bed cannot support graft survival, split-thickness skin grafting may offer a less invasive reconstructive option with lower surgical burden when an adequately vascularized recipient bed is present [20].

The distinctive feature of this case is not the use of NPWT or skin grafting in isolation, but the successful integration of these modalities into a staged limb-salvage strategy for extensive polymicrobial EG in a profoundly neutropenic patient, while preserving the extensor tendon paratenon and thereby avoiding flap reconstruction.

## Conclusions

Extensive ecthyma gangrenosum in profoundly neutropenic patients represents a major limb-salvage challenge requiring coordinated infection control, systemic recovery, and reconstructive planning. In this case, serial debridement followed by NPWT and delayed split-thickness skin grafting achieved successful limb preservation and functional recovery.

Careful preservation of the extensor tendon paratenon can maintain a vascularized recipient bed and expand reconstructive options in selected patients with extensive tendon-exposed wounds. A staged approach that prioritizes eradication of infection, optimization of the wound bed, and hematologic recovery may allow less invasive definitive coverage while avoiding flap reconstruction when adequate vascularity is preserved.
